# Supplementary material for: Predictors of Response and Survival in Immune Checkpoint Inhibitor-Treated Unresectable Hepatocellular Carcinoma
Source: Cancers (Basel). 2020 Jan 11;12(1):182. doi: 10.3390/cancers12010182 (PMC7017111; doi:10.3390/cancers12010182)
Supplement: Supplementary file 1 [file cancers-12-00182-s001.zip › Suppl. materials/Supp Tables.docx]

**Supplementary Table 1. Factors Associated with Best Disease Control in 90 HCC Patients with Evaluable Response**

| Characteristics |  | Univariate analysis | | |
| --- | --- | --- | --- | --- |
|  |  | OR | 95% CI | *p* value |
| Age, y | > 60 vs ≦ 60 | 0.769 | 0.316 – 1.872 | 0.563 |
| Sex | Male vs Female | 1.097 | 0.388 – 3.101 | 0.861 |
| HBsAg-positive | Yes vs No | 1.167 | 0.473 – 2.875 | 0.738 |
| Anti-HCV-positive | Yes vs No | 0.500 | 0.163 – 1.532 | 0.225 |
| Tumor size, cm | > 7 vs ≦ 7 | 0.643 | 0.258 – 1.599 | 0.342 |
| Tumor number | multiple vs single | 0.556 | 0.105 – 2.926 | 0.488 |
| Tumor shape | Infiltrative vs nodular | 1.536 | 0.599 – 3.940 | 0.372 |
| Tumor / Liver volume | >50% vs ≦50% | 0.882 | 0.340 – 2.289 | 0.797 |
| Portal vein invasion | Yes vs No | 1.046 | 0.443 – 2.467 | 0.919 |
| Main portal vein invasion | Yes vs No PVI | 0.955 | 0.297 – 3.073 | 0.938 |
| Portal branches invasion | Yes vs No PVI | 1.069 | 0.406 – 2.820 | 0.892 |
| Extrahepatic metastasis | Yes vs No | 0.473 | 0.197 – 1.133 | 0.093 |
| BCLC stage | Stage C vs B | 0.638 | 0.232 – 1.750 | 0.383 |
| AFP, ng/mL | > 400 vs ≦ 400 | 0.463 | 0.194 – 1.108 | 0.084 |
|  | < 10 vs ≧ 10 | 2.286 | 0.744 – 7.021 | 0.149 |
| NLR | > 2. 5 vs ≦ 2. 5 | 0.947 | 0.307 – 2.917 | 0.924 |
| Prothrombin time, INR | > 1.2 vs ≦ 1.2 | 0.852 | 0.326 – 2.227 | 0.743 |
| Platelet count | > 100K vs≦ 100K | 1.327 | 0.477 – 3.685 | 0.588 |
| ALT, U/L | > 40 vs ≦ 40 | 0.518 | 0.212 – 1.262 | 0.148 |
| AST, U/L | > 40 vs ≦ 40 | 0.485 | 0.196 – 1.202 | 0.118 |
| Ascites | Yes vs No | 0.598 | 0.241 – 1.485 | 0.268 |
| Child-Pugh class | Class B,C vs A | 0.387 | 0.128 – 1.166 | 0.092 |
| ALBI grade | Grade 2,3 vs 1 | 0.625 | 0.249 – 1.572 | 0.318 |
| Prior Sorafenib treatment | Yes vs No | 1.119 | 0.467 – 2.682 | 0.801 |
| Combined treatment* | Yes vs No | 2.746 | 0.794 – 9.499 | 0.111 |
| AFP reduction at 4^th^ week | Yes vs No | 13.056 | 4.299 – 39.652 | < 0.001 |
| Immunotherapy related AEs | Yes vs No | 0.914 | 0.273 – 3.066 | 0.885 |

Abbreviations:

AEs, adverse events; AFP, alpha fetoprotein; ALBI grade, albumin-bilirubin grade; ALT, alanine aminotransferase; AST, aspartate aminotransferase; BCLC stage, Barcelona-Clinic liver cancer stage;
CI, confidence interval; HBV, hepatitis B; HCV, hepatitis C; INR, international normalized ratio;
IO, immunotherapy; OR, odds ratio; NLR, neutrophil- lymphocyte ratio.

*Combined treatment: combined immune checkpoint inhibitors with tyrosine kinase inhibitors, including sorafenib, lenvatinib, and regorafenib.

^§^ Antibiotic exposure beyond prior 30 days: use of antibiotics in 30-60 days before immunotherapy but spare of antibiotics within 30 days before immunotherapy.
†AFP reduction at 4^th^ week: Baseline AFP > 10 ng/mL and AFP reduced > 10% from baseline serum level.

**Supplementary Table 2. Factors Associated with Overall Survival in 95 HCC Patients treated with immune checkpoint inhibitors**

|  |  | Univariate | | |  | Multivariate (Model 1)^#^ | | |  | Multivariate (Model 2)^#^ | | |
| --- | --- | --- | --- | --- | --- | --- | --- | --- | --- | --- | --- | --- |
|  |  | HR | 95% CI | *P* |  | HR | 95% CI | *P* |  | HR | 95% CI | *P* |
| Age, y | > 60 vs ≦ 60 | 1.252 | 0.676 – 2.318 | 0.476 |  |  |  | NA |  |  |  | NA |
| Sex | Male vs Female | 0.632 | 0.337 – 1.186 | 0.153 |  |  |  | NA |  |  |  | NA |
| HBsAg-positive | Yes vs No | 1.020 | 0.555 – 1.874 | 0.950 |  |  |  | NA |  |  |  | NA |
| Anti-HCV-positive | Yes vs No | 1.393 | 0.729 – 2.661 | 0.315 |  |  |  | NA |  |  |  | NA |
| Tumor size, cm | > 7 vs ≦ 7 | 2.450 | 1.362 – 4.409 | 0.003 |  |  |  | **NS** |  |  |  | **NS** |
| Tumor number | multiple vs single | 3.709 | 0.510 – 26.946 | 0.195 |  |  |  | NA |  |  |  | NA |
| Tumor / Liver volume | > 50% vs ≦ 50% | 2.425 | 1.323 – 4.444 | 0.004 |  |  |  | **NS** |  |  |  | **NS** |
| Portal vein invasion | Yes vs No | 1.829 | 1.008 – 3.321 | 0.047 |  |  |  | **NS** |  |  |  | **NS** |
| Extrahepatic metastasis | Yes vs No | 1.444 | 0.804 – 2.591 | 0.219 |  |  |  | NA |  |  |  | NA |
| BCLC stage | Stage C vs B | 1.854 | 0.828 – 4.154 | 0.134 |  |  |  | NA |  |  |  | NA |
| AFP, ng/mL | > 400 vs ≦ 400 | 2.039 | 1.102 – 3.773 | 0.023 |  |  |  | **NS** |  |  |  | **NS** |
|  | < 10 vs ≧ 10 | 0.255 | 0.079 – 0.826 | 0.023 |  |  |  | **NS** |  |  |  | **NS** |
| NLR | > 2. 5 vs ≦ 2. 5 | 1.010 | 0.467 – 2.185 | 0.981 |  |  |  | NA |  |  |  | NA |
| Prothrombin time, INR | > 1.2 vs ≦ 1.2 | 1.585 | 0.842 – 2.983 | 0.154 |  |  |  | **NS** |  |  |  | **NS** |
| Platelet count | > 100K vs≦ 100K | 0.928 | 0.479 – 1.799 | 0.825 |  |  |  | NA |  |  |  | NA |
| ALT, U/L | > 40 vs ≦ 40 | 2.463 | 1.370 – 4.428 | 0.003 |  |  |  | **NS** |  |  |  | **NS** |
| AST, U/L | > 40 vs ≦ 40 | 4.762 | 2.015 – 11.255 | < 0.001 |  | **4.034** | **1.305 – 12.471** | **0.015** |  | **4.033** | **1.316 – 12.360** | **0.015** |
| Ascites | Yes vs No | 2.782 | 1.551 – 4.989 | 0.001 |  |  |  | NA |  |  |  | **NS** |
| Child-Pugh class | Class A vs B | 0.260 | 0.143 – 0.472 | < 0.001 |  | **0.351** | **0.164 – 0.754** | **0.007** |  |  |  | NA |
| ALBI grade | Grade1 vs 2/3 | 0.189 | 0.079 – 0.453 | < 0.001 |  |  |  | NA |  | **0.252** | **0.091 – 0.695** | **0.008** |
| Prior Sorafenib treatment | Yes vs No | 0.952 | 0.528 – 1.717 | 0.870 |  |  |  | NA |  |  |  | NA |
| Combined treatment* | Yes vs No | 0.408 | 0.125 – 1.331 | 0.137 |  |  |  | **NS** |  |  |  | **NS** |
| Best tumor response | Progressive disease | 1 | – | – |  | 1 | – | – |  | 1 | – | – |
|  | Stable disease | 0.201 | 0.048 – 0.838 | 0.028 |  | **0.169** | **0.031 – 0.926** | **0.041** |  | **0.132** | **0.023 – 0.779** | **0.025** |
|  | Objective response | 0.157 | 0.055 – 0.443 | < 0.001 |  | **0.107** | **0.032 – 0.358** | **< 0.001** |  | **0.127** | **0.036 – 0.451** | **0.001** |
| AFP reduction at 4^th^ week | Yes vs No | 0.372 | 0.172 – 0.809 | 0.013 |  |  |  | **NS** |  |  |  | **NS** |
| Immunotherapy related AEs | Yes vs No | 0.746 | 0.294 – 1.893 | 0.537 |  |  |  | NA |  |  |  | NA |

Abbreviations: ALBI grade, albumin-bilirubin grade; AEs, adverse events; AFP, alpha fetoprotein; AL(S)T, alanine(aspartate) aminotransferase; BCLC stage, Barcelona-Clinic liver cancer stage; CI, confidence interval; HB(C)V, hepatitis B(C); HR, hazard ratio; INR, international normalized ratio; NA, not adopted; NLR, neutrophil- lymphocyte ratio; NS, not significant.

* Combined treatment: combined immune checkpoint inhibitors with tyrosine kinase inhibitors, including sorafenib, lenvatinib, and regorafenib.

^#^ Model 1 enrolled parameters with p value < 0.2 in univariate analysis into multivariate analysis, except ascites and ALBI grade.

^#^ Model 2 enrolled parameters with p value < 0.2 in univariate analysis into multivariate analysis, except Child-Pugh class
