# Supplementary figures and images for: Predictors of Response and Survival in Immune Checkpoint Inhibitor-Treated Unresectable Hepatocellular Carcinoma
Source: Cancers (Basel). 2020 Jan 11;12(1):182. doi: 10.3390/cancers12010182 (PMC7017111; doi:10.3390/cancers12010182)

Supplementary figure 1

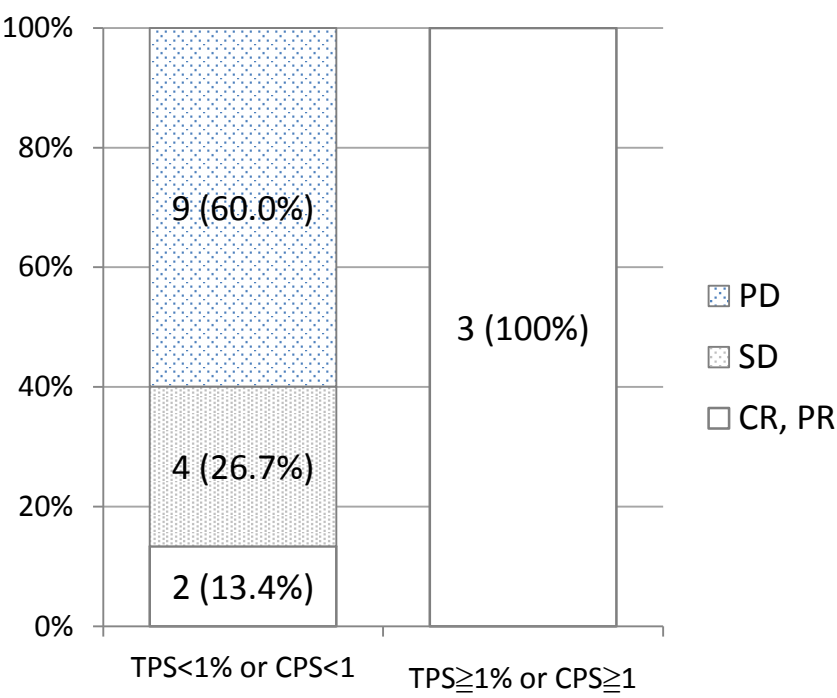

| TPS or CPS | TPS<1% or<br>CPS < 1 | TPS ≥ 1% or<br>CPS ≥ 1 |
|------------|----------------------|------------------------|
|            |                      |                        |
| CR, PR, SD | 6                    | 3                      |
| PD         | 9                    | 0                      |

*p* = 0.206

Evaluable PD-L1 level, n = 18

Supplement: Supplementary file 1 [file cancers-12-00182-s001.zip › Suppl. materials/Supp Fig 1. PD-L1 & BR.pdf]

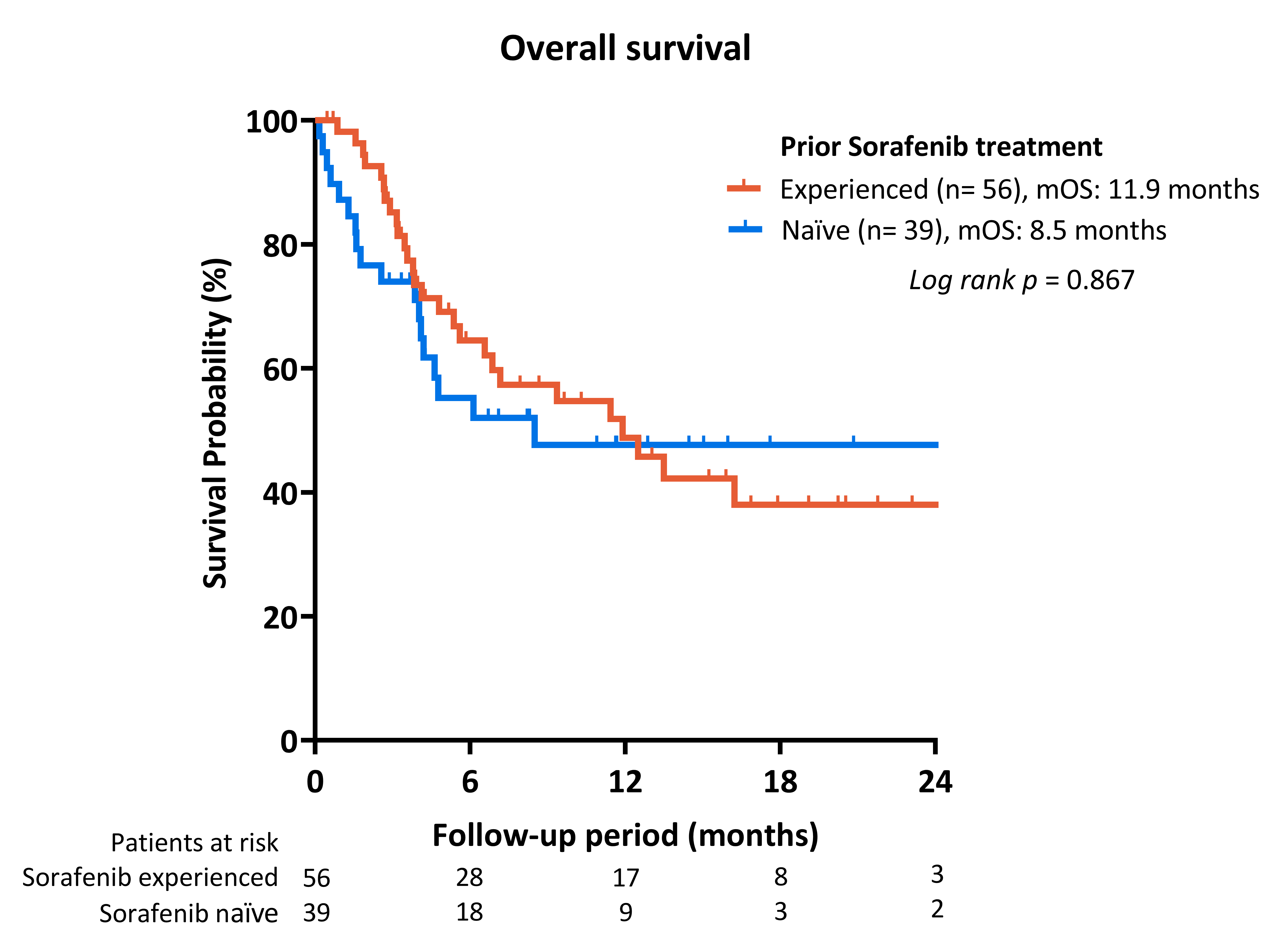

Supplement: Supplementary file 1 [file cancers-12-00182-s001.zip › Suppl. materials/Suppl Fig 2. PreSora-OS.tif]
